# Electronic Skin Wearable Sensors for Detecting Lumbar–Pelvic Movements

**DOI:** 10.3390/s20051510

**Published:** 2020-03-09

**Authors:** Yuxin Zhang, Pari Delir Haghighi, Frada Burstein, Lim Wei Yap, Wenlong Cheng, Lina Yao, Flavia Cicuttini

**Affiliations:** 1 Faculty of Information Technology, Monash University, Melbourne, VIC 3145, Australia; yuxin.zhang@monash.edu (Y.Z.); pari.delir.haghighi@monash.edu (P.D.H.); 2 Department of Chemical Engineering, Monash University, Melbourne, VIC 3800, Australia; lim.yap@monash.edu; 3 School of Computer Science and Engineering, The University of New South Wales, Sydney, NSW 2052, Australia; theresa0125@gmail.com; 4 Department of Epidemiology and Preventive Medicine, School of Public Health and Preventive Medicine, Monash University, Melbourne, VIC 3004, Australia; flavia.cicuttini@monash.edu

**Keywords:** E-Skin sensors, movement detection, wireless sensing technology, monitoring

## Abstract

Background: A nanomaterial-based electronic-skin (E-Skin) wearable sensor has been successfully used for detecting and measuring body movements such as finger movement and foot pressure. The ultrathin and highly sensitive characteristics of E-Skin sensor make it a suitable alternative for continuously out-of-hospital lumbar–pelvic movement (LPM) monitoring. Monitoring these movements can help medical experts better understand individuals’ low back pain experience. However, there is a lack of prior studies in this research area. Therefore, this paper explores the potential of E-Skin sensors to detect and measure the anatomical angles of lumbar–pelvic movements by building a linear relationship model to compare its performance to clinically validated inertial measurement unit (IMU)-based sensing system (ViMove). Methods: The paper first presents a review and classification of existing wireless sensing technologies for monitoring of body movements, and then it describes a series of experiments performed with E-Skin sensors for detecting five standard LPMs including flexion, extension, pelvic tilt, lateral flexion, and rotation, and measure their anatomical angles. The outputs of both E-Skin and ViMove sensors were recorded during each experiment and further analysed to build the comparative models to evaluate the performance of detecting and measuring LPMs. Results: E-Skin sensor outputs showed a persistently repeating pattern for each movement. Due to the ability to sense minor skin deformation by E-skin sensor, its reaction time in detecting lumbar–pelvic movement is quicker than ViMove by ~1 s. Conclusions: E-Skin sensors offer new capabilities for detecting and measuring lumbar–pelvic movements. They have lower cost compared to commercially available IMU-based systems and their non-invasive highly stretchable characteristic makes them more comfortable for long-term use. These features make them a suitable sensing technology for developing continuous, out-of-hospital real-time monitoring and management systems for individuals with low back pain.

## 1. Introduction

Low back pain (LBP) is the leading cause of disability among individuals [1,2]. Lumbar–pelvic movements have been identified as major risk factors for developing LBP as well as indicators of LBP rehabilitation progress [3,4,5]. Assessment of how individuals perform lumbar–pelvic movements during the rehabilitation progress could assist with identifying functional limitations and physical predictors of low back pain, and monitoring treatment outcomes [4]. The ability to assess these movements in a non-invasive and sensitive way in real-time and out-of-hospital could improve LBP prevention and treatment [3,4]. According to recent studies [6,7], there are significant differences between individuals with and without LBP for how the same lumbar–pelvic movement is performed, based on speed, angle range (range of movement), acceleration, and intensity. These features can be used to characterise each lumbar–pelvic movement for individuals with and without LBP.

Assessment of the anatomical angle such as the angle of back extension or flexion is essential for the detection and measurement of lumbar–pelvic movements. Recent growth and advances in mobile and wireless sensing technologies present an unprecedented opportunity for collecting physiological signals and body movement data in mobile healthcare and diagnostic medicine. Due to this advancement, a number of sensing, systems including vision-based systems [8] and IMUs-based systems that can monitor the angle of body movements, have emerged in the market [9]. One common characteristic of these systems is to compute the angle by sensing coordinates of different points on a human body in a three-dimensional space. This data can also be used to calculate the speed, acceleration, and direction of the angle. Vision-based systems can provide precise and accurate measurements, but their use is limited to the laboratory setting. Additionally, their costs are relatively high. The IMU-based systems can be used for out-of-hospital monitoring. Yet, they require the application of multiple IMUs to provide a relatively accurate and precise measurement. They also need wired or wireless modules for the communication of each IMU. This can lead to a bulky and rigid packaging, which can be very uncomfortable for individuals to wear over a long period of time. Moreover, the accuracy and precision of measurement of the gyroscope in these systems cannot be guaranteed if the speed of movement is very slow.

Electronic-skin (E-Skin) wearable sensors [10] have recently gained popularity due to their ultrathin, flexible, stretchable, self-healing, skin mimicking characteristics which enables them to perform mechanical, chemical, and biophysiological sensing while also being able to attach comfortably to human skin [10,11,12]. A plethora of materials, such as conductive polymers, metallic nanomaterials, ionic liquids, liquid metals, and conductive textile, have been exploited by researchers in fabrication of novel electronic skins [13,14]. Among the materials mentioned above, high aspect ratio one-dimension (1D) synthetic nanomaterials (such as metallic nanowire, metal oxide nanowire, and carbon nanotubes [14,15]) could be used to develop highly sensitive and highly stretchable soft electronic skin wearable strain gauge sensor [16,17,18,19]. A number of demonstrations on the capability of these high sensitivity nanomaterial-based E-Skin wearable sensors have been reportedly used for monitoring pulse signal, detecting throat, finger, and ankle movement [13,16,17,18]. Since these sensors can detect minor strains and skin deformations [16,17,18,19], they are not only capable of measuring the angle of anatomical joints, but also of measuring minor movements of human body that cannot be detected by MEMS-based sensors such as inertial measurement unit (IMU). With the ability to attach conformally to human skin, E-Skin sensors can be used to detect subtle human motions such as lumbar–pelvic movements [19,20,21,22]. Lumbar–pelvic movements consist of flexion, extension, anterior posterior pelvic tilt, lateral flexion, and rotation. Detecting and measuring these movements can significantly help medical experts to collect detailed information to understand the relationship between physical movements and low back pain from a personalised perspective.

This paper investigates the feasibility of using the state-of-the-art E-Skin sensors to detect five standard lumbar–pelvic movements including flexion, extension, pelvic tilt, lateral flexion, and rotation, and measure the change of angles during each movement. Compared to vision-based and IMU-based systems, E-Skin sensors are more comfortable to wear and offer a higher sensitivity to precisely detect subtle movements [22]. However, there is limited research reported on the use and validation of E-skin sensors for detecting and measuring lumbar–pelvic movements. In this paper, we introduce a method to measure and analyse the patterns of E-Skin sensor outputs (i.e., skin deformation data) for detecting the lumbar–pelvic movements. To the best of our knowledge, we are the first to carry out a detailed experimental study on using E-Skin sensors for lumbar–pelvic movement detection and measuring. The E-Skin sensor we used has been developed by the Monash University NanoBionics Group (Melbourne, VIC, Australia) [20]. To validate the performance of E-Skin sensor on detecting and measuring lumbar–pelvic movements, ViMove (DorsaVi, Melbourne, VIC, Australia) was used as a golden standard. In previous report [6], 34 participants, including 18 with LBP and 16 without LBP, were recruited to perform different lumbar–pelvic movements with both ViMove and Vicon systems. The results showed that there was a clinically acceptable level of agreement between these two systems in terms of measuring lumbar–pelvic movements.

The contributions of this paper can be summarised as the following: (1) a comprehensive review and comparative analysis of existing sensing technologies and systems for measuring and detecting lumbar–pelvic movements, (2) introducing a method to measure and detect lumbar–pelvic movements including flexion, extension, anterior posterior pelvic tilt, lateral flexion, and rotation, and their anatomical angles by using E-Skin sensors, and (3) providing recommendations for future research based on our experimental study.

This paper is organised as follows. In Section 2, existing wireless sensing technologies-based systems, which can be used for monitoring lumbar–pelvic movements, are reviewed based on our classification of existing sensing technologies. In Section 3, the design of experiments is described. In Section 4, results of the exploratory experiment are discussed. In Section 5, the conclusion is presented.

## 2. Classification of Sensing Technologies for Body Motion Detection

Existing sensing technologies for detecting and measuring body movements can be classified into three main categories: vision-based systems, IMUs, and flexible sensors.

### 2.1. Vision-Based Sensing Systems

Vision-based systems normally consist of high-speed cameras and reflection markers or 3D cameras [6]. Their operation highly depends on adjusting camera settings, selecting proper lighting conditions, and the use of video/image processing algorithms. Vision-based systems can be further divided into two categories of marker-based systems and marker-less systems.

#### 2.1.1. Marker-Based System

Marker-based systems require reflection or transmitting markers for the measurement of physical movement. It uses infrared optical or high-speed cameras to detect the light reflection of the markers and measures activity using the computation of the markers’ trajectories in a three-dimensional space [23]. Based on different settings (e.g., the camera numbers and marker protocols), the accuracy of the marker-based system can be close to 0.1 mm and the sampling frequency can be as high as 1000 Hz [24]. This type of systems have been used for many studies such as measuring hand movements [25] and robotic motion monitoring [26].

Vicon is one of the world-renowned vision-based motion tracking systems which has been used in a variety of body motion related research and clinical studies, such as [8,27]. The advantage of the Vicon system is its ability to precisely reconstruct physical movements using markers. In [28], the Vicon system is proved to have a high accuracy in terms of measuring spinal and hip mobility. It is also the gold standard used to validate the performance of other motion tracking systems, including inertial measurement unit (IMU)-based measurement systems and mark-less vison systems [6].

Similar products have also been utilised for studies related to different physical movements, such as Optotrak Certus motion tracking system [29], MotionAnalysis system [30], and CODA motion analysis system [31].

Generally, the functions of these commercialised marker-based vision systems are similar. These systems provide a clinical-level solution for real-time measurement of physical activity. The high precision of the measurement outperforms other systems, but they have a number of limitations. Firstly, the hardware and software are expensive. Secondly, installation of the system is time-consuming and complicated, particularly for the calibration of the camera position and light condition. Thirdly, it requires laboratory settings, which limit its use in measuring daily activities. Fourthly, the soft tissue artefacts, including incorrect marker positioning and skin sliding over the bones, may affect the measurement accuracy of marker-based systems [23].

#### 2.1.2. Marker-Less System

Marker-less vision-based systems use multi-cameras, IR sensors, or RGB-D cameras in the analysis of body movements or postures. The analyses could be done with a single image or video clips and do not require the attachment of markers to individuals.

Microsoft Kinect is a well-known commercialised marker-less motion-capturing system [32]. It consists of a wide-angle motion sensing camera with an IR sensor to construct the depth information of the image/video. It is primarily designed for tracking joint movements of the human body [33]. Nevertheless, the performance of Kinect is limited by its latency and tracking range [34].

The organic motion system is a similar mark-less vision system with a wide tracking range compared to Kinect [33]. KinaTrax is another powerful marker-less vision-based posture analysis system with multiple camera set-ups that can adequately cover an area size of a baseball court [35].

In general, the marker-less based systems are relatively cheaper and easier to setup than the marker-based systems [35]. However, they provide lower accuracy for real-time movement tracking. Additionally, the real-time processing performance of the marker-less system is also not as good as the other sensing systems such as IMU-based systems [36]. With the development of the RGB-D cameras and image/video processing technology, the marker-less based vision systems may capture more complex movements and postures analysis in the future. Currently, these systems need sophisticated lighting conditions to achieve a better performance, so they are not suitable for continuous monitoring in different environments [36].

### 2.2. Inertial Measurement Unit (IMU)

IMU consists of accelerometers, gyroscopes, and occasionally magnetometers [9]. With the development of microelectronic technology, these sensors have become smaller and easier to wear with high accuracy of measuring body movements [37]. Based on the number and type of the sensors, they can be further classified into IMU sensor systems and garment integrated sensor systems. IMU sensor systems include single and dual IMU-based systems. The systems which contain more than two IMU sensors are categorised under the multiple sensory systems.

#### 2.2.1. IMU Sensor Systems

Single IMU sensor-based systems are mostly designed to monitor the posture and movement of an individual by placing the sensor on the waist or lower back area [38]. Most of these systems are bio-feedback systems, such as Spineangel [39], Lumo Lift [40], and Upright posture trainer [41], designed to help improve the awareness of individuals of their sitting posture or inappropriate movements. One of their limitations is the difficulty in measuring complex physical movements and generating detailed information. Thus, combining IMU sensor with Vision-based sensors has become necessary for real-time sports training tasks [36].

Unlike the single IMU sensor system, the dual IMU sensor-based systems enhance the possibility of measuring complex movements. Considering that the single IMU device is a single point that indicates the lumbar–pelvic area, it can only reflect a single point movement in three-dimensional spaces. However, the lumbar–pelvic area is a soft surface which moves simultaneously with the spine. Thus, the movement of the two attached points in the dual IMU sensor-based device can be used to simulate the movement of the spine and indicate the movement of the lumbar–pelvic area.

Several dual IMU sensor-based systems, designed to measure the lumbar–pelvic movements, exist. Most of these systems developed for rehabilitation purposes include ViMove [42], Valedo Motion [43], and RIABLO [44].

ViMove is a commercialised movement monitoring device developed by DorsaVi [42]. It consists of two IMU sensors, two surface EMG sensors, and one remote recording and feedback device (RFD). It can measure multiple movements including the lumbar–pelvic area. Additionally, studies have been conducted on the concurrent validity of ViMove for measuring lumbar region inclination motion compared with Vicon [6]. ViMove is also used in several other lower back pain related clinical trials [45,46]. In contrast to Valedo and RIABLO, ViMove offers a means of monitoring daily activities, which highly enhances its mobility. The raw data of the sensors can be accessed through its application. Yet, it does not provide direct access to the data captured through APIs in real-time.

In general, the single IMU sensor-based systems are useful for monitoring spinal postures and lumbar–pelvic movements. By using different advanced algorithms, the IMU can process human motion data in real-time with good accuracy [47]. Although, numbers of studies have proposed solution for solving the drift issues in IMU system, the drift error still exists due to the nature of IMU [48,49,50]. The dual IMU sensor-based systems are highly effective in monitoring lumbar–pelvic movements. Although these systems do not require complex laboratory settings like the vision-based systems, they still provide accurate measurement. Presently, the dual IMU sensor-based system is preferable because of its ability to monitor and measure complex lumbar–pelvic movements. Nonetheless, they are uncomfortable for long-time use because of their weight and rigidity.

#### 2.2.2. Multiple Sensory System (Garment Integrated Sensor System)

Multiple Sensory systems (Garment integrated sensor systems) use different types of sensors such as temperature, IMU, ECG, or EMG sensors, which are integrated into garments (e.g., tops, vests, or sportswear). The integration of these sensors usually creates a small wireless network, known as body sensor network (BSN), body area network (BAN), or wireless body area network (WBAN). The IMUs are the sensors, which are used to detect and measure physical movements.

The multi-IMU sensor-based garment systems are designed for the measurement of three-dimensional human movement. They commonly have 8 to 12 IMU sensor nodes attached to garments and located on different parts of the human body including the head, shoulder, elbow, wrist, waist, knee, and foot [51,52,53]. Since these systems contain multiple nodes, they can measure different physical activities based on the movements of different body parts. For example, Zishi is a garment-based sensing system for trunk posture monitoring [54]. The garment is integrated with accelerometers and gyroscopes which make it capable of detect static and dynamic trunk movements with high accuracy (<1.5 degree). However, these garment integrated sensors are expensive, non-washable, and difficult to structure because of the quantity of the IMU sensors [53]. Furthermore, their design has to be personalised for different individuals such as different height and body measures.

The purpose of garment sensing systems is to use multiple sensors together without compromising mobility. The advantage is their capacity for data fusion from distinct types of sensors which gather different data. However, the sweat and body movements from the daily use of garments may trigger repositioning of the garment and cause errors in the measurement. The calibration of this type of system is also very complicated [55].

### 2.3. Flexible Sensors

Flexible sensors refer to sensors which can be flexed or stretched while the body moves. The flexible sensors are small and easy to integrate with a garment or other skin-contact substrates. There are two types of flexible sensors. One is plastic optical fibre sensor, which has been used in spine posture measurements [56,57]. The mechanism of these sensors is to monitor the bend of the structural beams. However, the plastic optical fibre is not soft enough to be attached to the human body and move as the body moves. Therefore, it is better for measuring anatomical joint movements rather than detecting complex movements such as lumbar–pelvic movements.

The other type flexible sensors are the resistive flex sensors [58]. The principle of resistive flex sensors is variable resistive characteristics of a conductive material [59]. This type of sensor consists of three components: the contact, sensor film, and substrate [60,61]. The contact is connected to the circuit board which detects the changing current. The sensor film is made of a conductive material. The resistance changes with the deformation of the conductive material. The substrate is the middle layer between sensor film and human skin. The intrinsic properties of the substrate determine the flexibility of the sensor. The E-Skin sensor is part of the resistive flex sensors. With the advance in nanotechnology, the sensitivity and stretchability of conductive material has been significantly improved. E-Skin sensors are now capable of measuring the slightest movement of the human body. With flexible integrated circuit boards (such as central processing unit and Bluetooth component), the sensory data can be wirelessly transmitted to a smartphone for further analysis [62]. Compared to the IMUs and Vision-based systems, the E-Skin sensor is cheaper, softer, and smaller, which makes it a suitable alternative for detecting and measuring physical movements. Because of its high sensitivity, most of studies have used E-Skin sensors to measure subtle body movements such as the movement of fingers [63,64]. Additionally, human skin is not a plane surface, but the E-Skin sensor can be easily attached to human skin unlike other existing sensors. Therefore, the soft tissue artefacts can be avoided by using E-Skin sensor.

**Figure 1:**
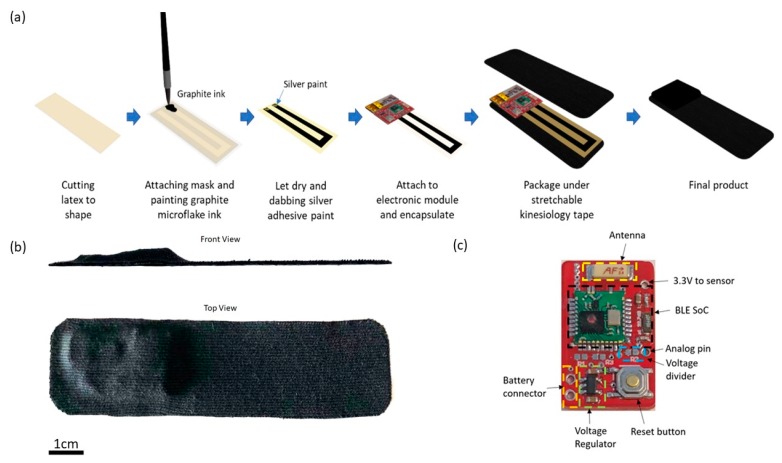
(**a**) Schematic Illustration of E-Skin Sensor Fabrication; (**b**) E-Skin Sensor Dimension; (**c**) E-Skin Electronic Module Description.

The commercially available stretch sensor, StretchSense, is a flexible strain gauge sensor like the E-Skin sensor we used in this study [65]. However, the StretchSense sensor utilizes capacitive strain sensing to measure the degree of strain [66]. Capacitive-based strain gauge sensor has a 5-layered sandwich structure, a dielectric layer sandwiched between two electrode layers and protected by two protective layers [66]. Compared to StretchSense, the E-Skin sensor utilizes piezoresistive strain sensing that consists of a 3-layered structure, in which a stretchable conductive layer is protected by two protective layers (see Figure 1). Therefore, the E-Skin sensor is much thinner than the Stretch sense sensor and more comfortable to wear, with better accuracy and higher sensitivity. Additionally, the E-Skin sensor measures the change in electrical resistance so the circuit could be designed simpler compared to stretch sensors. It only needs a micro-control unit (MCU) with a 12-bit Analog-digital convertor (ADC) to read the changes in resistance. In [67], a multichannel conductive liquid-based strain sensor has been introduced. The results show the good performance (mean absolute errors <8 degree) in tracking joint angles during the gait cycles. However, the conductive liquid-based sensors may suffer from leakage problems, which makes them not suitable for wearable applications. The thickness of conductive liquid-based sensors is also much higher than E-Skin sensor. Moreover, it needs to be mounted on a sensing suit for motion measurement, which makes it less accurate comparing to the sensors which are directly attached on human body. Then, [68] introduced a similar strain sensor for human motion detection. Unlike E-Skin sensor, it uses a stretchable carbon nanotube to measure the 1D strain. The results show it is capable of detecting different human motions, but the performance of measurement was not reported. Additionally, this type of sensors also needs to be mounted on stockings or a sensing suit.

### 2.4. Summary

Based on the review presented, it appears that vision-based systems provide the most accurate and precise monitoring tool for measuring human body motion. However, they are very expensive, and require proper camera settings and lighting conditions [27]. They can be only used in a lab environment, which limits their use for continuous out-of-hospital monitoring. IMU sensor-based systems are the most common used sensors for out-of-lab physical motion monitoring [69]. IMU sensors need to be attached to human body using stickers. However, the packaging material of the sensors are usually made of a rigid plastic that makes them inflexible and bulky [46]. The deformation of the skin may affect the repositioning of stickers when an individual performs physical activities, and this could lead to an inaccurate measurement [70]. Additionally, for monitoring lumbar–pelvic movements, the placement of the sensor is around the waist of individual. The belt or edge of the clothes (such as jean trousers) has the potential to affect the measurement if it touches the sensors during the movements. To achieve accurate results, normally it is necessary to use at least two sensors to detect complex body movements such as lumbar–pelvic movements [42]. Therefore, the IMU sensor-based system can be problematic for long-term monitoring.

Garment-integrated sensing systems are another option to attach multiple sensors on human body. However, they are heavy and uncomfortable to wear for a long period of time and are usually non-washable [71]. They can also suffer from the same repositioning problem that stickers have when an individual performs movements [53].

E-Skin sensors (resistive flex sensors) provide a suitable solution for detecting and measuring lumbar–pelvic movements. Compared to vision-based systems, they are much cheaper, and do not require a great deal of effort in setting up the equipment. Unlike other flexible sensors [57], they are soft and stretchable enough to attach to any human skin surface conformally. This reduces the repositioning problem of IMU sensors. They can also detect the slightest movement of the skin which can be used to determine the movement and subcomponents of the movements. Based on this comparative analysis, we concluded that E-Skin sensors have a great potential for out-of-lab lumbar–pelvic movement detection and measuring which is a focus of this paper. A detailed comparison of existing sensing systems for human motion detection and measurement is given in Table 1.

## 3. Materials and Methods

We have designed and fabricated E-Skin sensors for detecting and measuring all the five types of lumbar–pelvic movements, including flexion, extension, pelvic tilt, lateral flexion, and rotation. The following subsections discuss the details of these experiments.

This research has been approved by Monash University Human Research Ethics Committee.

### 3.1. Instrumentation and Data Collection

E-Skin sensors are fabricated based on a graphite microflake hybrid conductive network-based strain sensor and the illustration of the fabrication process is shown in Figure 1a. In short, copper nanowires ink and graphite microflake ink were formulated and painted in U-shape on latex substrate. A silver paste is applied on both ends of the U-shaped strain sensor and attached to the electronic module. Lastly, the strain sensor is encapsulated with liquid latex and the whole device is packaged between two layers of Elastoplast stretchable sports kinesiology tape. The sports kinesiology tape is hypoallergenic and can be directly attached to human skin. The length and width of the fabricated device is 10 cm and 2.5 cm, respectively, with the maximum thickness of the device being 0.5 cm as shown in Figure 1b.

As shown in Figure 1c, the core of the electronic module is an nRF51822 Bluetooth Low Energy (BLE) system-on-chip from Nordic semiconductor [72]. A voltage regulator is used to regulate input voltage to 3.3 V to power the BLE SoC (System on a Chip) and the strain sensor. The other terminal of the strain sensor is connected to the analog pin of the BLE SoC.

**Figure 2:**
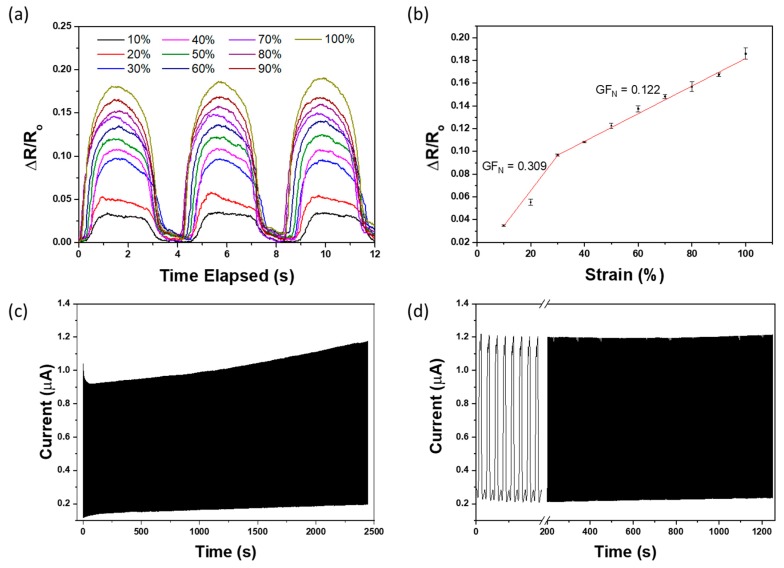
(**a**) Plot of resistance change of E-skin sensor under an applied strain in the range of 10% to 100% with increment of 10% strain (**b**) Strain-response plots for the E-skin sensor. (**c**) Durability test of 1000 cycles 10% strain at a frequency of 0.4 Hz and (**d**) the performance of the strain sensor after 1000 strain cycles.

The mechanical property of the fabricated E-skin sensor was studied by straining the sensor from 10% to 100% with increment of 10% using Thorlabs’ motorised linear translation stage and the change in electrical resistance was measured and recorded using VersaSTAT 4 Potentiostat Galvanostat. As shown in Figure 2a, the signal recorded upon straining the E-skin sensor is relatively consistent. The strain-resistance response curve for E-skin sensor under different range of physical strain was also investigated and plotted in Figure 2b. The gauge factor (GF) of the E-skin sensor was calculated using Equation (1), where R is the electrical resistance of the E-skin recorded upon stretching, R_o_ is the initial electrical resistance of E-skin sensor at rest, and ɛ is the degree of strain applied to E-skin sensor. The gauge factor was calculated based on the data plotted in Figure 2a. The gauge factor of the strain sensor divided into two sections. At the lower strain of 10% to 30%, the gauge factor of the sensor was found to be 0.309 whereas at a higher strain of 30% to 100%, the gauge factor was 0.122, which is less sensitive than the E-skin sensor at lower strain. Based on the gauge factor calculated, the E-skin sensor was found to have the ability of measuring the strain at a wide range of 10% to 100%, which is particularly useful for application such as monitoring bending angle of human body posture [45,46]. The durability of the E-Skin sensors was tested by conducting 1000 cycles of strain test at 30% strain, which is shown in Figure 2c. The outputs of the sensor fluctuate slightly in the initial stage and stabilised after 1000 cycles of strain. After 1000 cycles, the E-Skin sensor performance remained stable and was able to fully return to its original value when being released after strain (shown in Figure 2d), which proves its durability and reliability. Hence, prior to the trial, the freshly fabricated E-Skin sensors will always be pre-strained for 1000 cycles as a “learning” process for the E-Skin sensors. Since the E-Skin sensor uses graphite microflakes hybrid conductive network, its performance will not be affected by any chemical reactions such as oxidation during the lumbar–pelvic movement detection [73]. However, to ensure the sensing accuracy, each E-Skin sensor was only used once on each participant for one experiment session.

(1) $$GF=\frac{1}{\varepsilon }\cdot \frac{R-R_{0}}{R_{0}}$$

**Figure 3:**
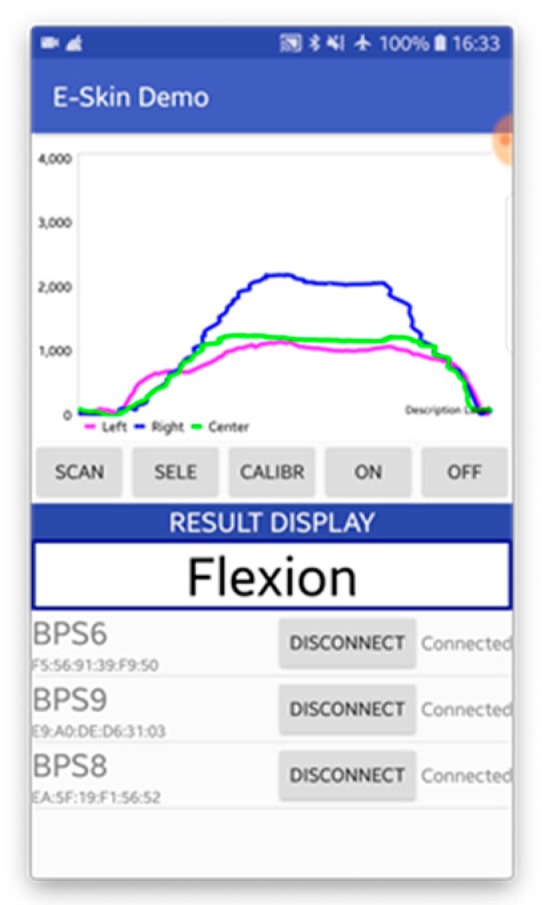
Data Collection Mobile Application User Interface.

The E-Skin sensor data is collected wirelessly via smartphone, and the sample rate of 15 Hz for each E-Skin sensor to fit the throughput of smartphone BLE transmission. The user-interface of the data collection mobile application is shown in Figure 3. This application can connect to 3 E-Skin sensors at the same time. The main functions of this application include data transmission control module, data processing module, local storage control module, and real-time data visualisation module. The data transmission control module contains two commands: start/end data transmission and E-Skin sensor labelling. The user can use these two commands to directly control all connected E-Skin sensors to start and end the data transmission simultaneously and label different connected E-Skin sensor with user-designated name such as left, right, and centre. Once the application receives the data streams (including timestamp and data value), data processing module allocates each data stream from different E-Skin sensor into separated buffers (The length of the data buffer is set to be 15 bytes based on the sample rate) based on the Bluetooth identifiers (UUID). For this android application a simple Kalman filter Java class was implemented for data smoothing [74]. Based on the empirical experiences, suitable values for the initiation of the filter were: measurement uncertainty (r) = 4, estimate uncertainty (p) = 4, process variance (q) = 0.05. The current estimation and estimate uncertainty update were calculated based on the following formulas, where k stands for Kalman gain.

(2) $$k=\frac{p}{\left(p+r\right)}$$

(3) $$\begin{array}{c} current\;estimation\\ \;\;\;\;\;\;\;=last\;estimation+k*\left(measurement-last\;estimation\right) \end{array}$$

(4) p=(1−k)∗p+ |last estimation−current estimation|∗q

Then the processed data buffers are sent to the local storage control module and real-time data visualisation module, respectively. The local storage control module creates text files for each data stream and stores the timestamps as well as the corresponding data values. The real-time data visualisation module displays the processed data in a dynamic line chart with user-designated labels.

**Figure 4:**
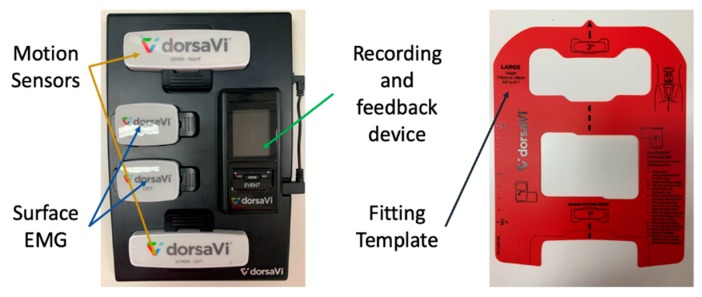
ViMove System Components.

ViMove (DorsaVi, Melbourne, Australia) was used as a golden standard for the evaluation of E-Skin sensors’ performance on lumbar–pelvic movement detection. ViMove is a clinically validated instrument for monitoring lower back movements [6]. As shown in Figure 4, ViMove system consists of 4 parts: the upper and lower motion sensors (located at L1 and PSIS), the left and right surface EMG sensors (located at each side of the spine around L3), recording and feedback device and monitoring software programs. The ViMove suite also includes a low back fitting template to help the user to easily position the sensors on the body according to their height. The template is also shown in Figure 4. The EMG sensors are used to assess the muscle activities, and therefore, in our experiments we did not use these sensors. The sample rate of ViMove is 20 Hz. The outputs of ViMove can be collected using ViMove data collection software on a PC. Table 2 compares E-Skin to ViMove based on seven criteria.

### 3.2. Study Design and Experimental Procedure

**Figure 5:**
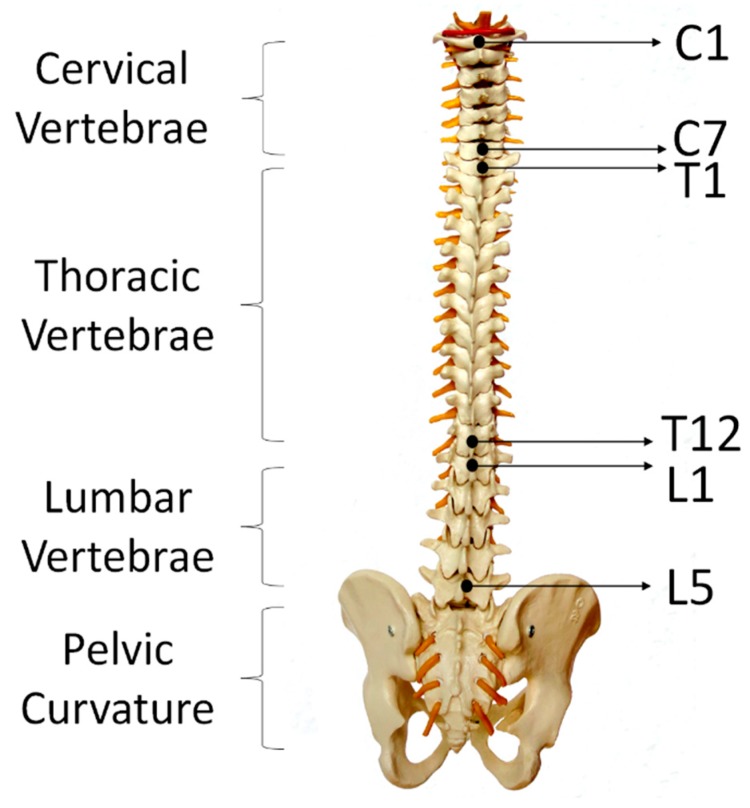
Regions of Spine.

**Figure 6:**
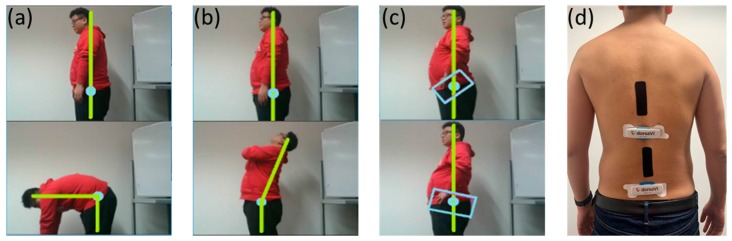
Experiment Description: (**a**) Flexion; (**b**) Extension; (**c**) Pelvic Tilt; (**d**) Sensor Placement.

Lumbar–pelvic movements refer to the lower back area movements that include flexion, extension, lateral flexion, anterior posterior pelvic tilt, and rotation. These movements can be performed by individuals during different physical activities. 6 participants (including 2 males and 4 females aged 22–30 years with height 159–183 cm, weight 47–120 kg, and 2 of them have LBP history in the past 6 months) were recruited in this experiment as our aim initially was to analyse skin deformation data and compare to ViMove data in order to develop a method for detecting the five lumbar–pelvic movements. The participants were instructed to perform the five standard lumbar–pelvic movements based on the low back pain patients’ rehabilitation assessment process [5]. These five lumbar–pelvic movements follow the biomechanical standards and widely used by medical experts to assess the lumbar movements performed by different individuals [75]). Each participant performed each movement for 5 times and repeated the entire experiment for 3 times. After performing each lumbar–pelvic movement (LPM), participants had to return to their static position (i.e., standing in a relaxed manner) for a few seconds before continuing to do the next movement. Participants could choose to withdraw or stop to rest at any time during the experiments. Altogether, we have collected 15 times of each lumbar–pelvic movements. In order to make the experiment comparable to real life scenario, participants were instructed to perform the LPMs at any speed with any bending or turning angles. We had utilised the regions of spine as human back landmarks to indicate the locations of sensor placement. As shown in Figure 5, adults have 24 separate vertebrae including 7 cervical vertebrae from the neck (C1–C7), 12 thoracic vertebrae from the upper back (T1–T12) and 5 lumbar vertebrae from the lower back (L1–L5). The top E-Skin sensor was attached vertically around T3–T7 and the bottom one was attached vertically around L1–L5 as shown in Figure 6d. The ViMove lower motion sensor was attached along the line of posterior superior iliac spine (PSIS). PSIS of the participant was measured by palpation. The placement of the ViMove motion sensors were based on the ViMove templates. The placement of E-Skin sensor was informed by the consultation with the medical experts involved in this research. This sensor placement has been identified to produce the maximum E-Skin sensor output during the performance of flexion, extension and pelvic tilts. In this paper, we present our analysis with one participant because there is no significant difference between participants with and without LBP in terms of the lumbar–pelvic movement data pattern [76]. The participant was instructed to repeat each movement 5 times at the same speed as possible. Figure 6 (a–c) show the flexion, extension, and anterior posterior pelvic tilt, respectively.

ViMove and E-Skin sensors use two different sampling frequency. The frequency of ViMove data is generally slightly lower than 20 Hz (19.0–19.8 Hz), and the E-Skin sampling rate is around 15 Hz. In order to compare the outputs of these two sensors, we calculated the average of data points per second for each sensor. For example, the ViMove collected 19 data points in 1 s and E-Skin sensor A, B, C received 14, 15, and 16 data points in the same second, respectively. We summed all data points in this second for each sensor and divided the sums by the numbers of the data points of each sensor. This allowed us to compare their average values using the same timestamp. Since the participants were instructed to perform movements slowly, this downsampling procedure would not affect the subsequent analysis.

### 3.3. Results Analysis

During our experiments, five sets of output sensor data was collected from both ViMove and E-Skin simultaneously while the participant was performing flexion, extension, pelvic tilt, rotation, and lateral flexion (See Figure 6d).

The E-Skin sensor raw signal output range from 0 to 4096 Analog-to-Digital Units (ADU) [77]. The value decreases when the E-Skin stretches. In order to clearly depict the changes in E-Skin outputs, we converted the absolute E-Skin output into the change in electrical resistance for the E-Skin sensor for comparison rather than the raw signal output. The absolute E-Skin output was calculated by using E-Skin raw signal output to determine a baseline value. The baseline value was calculated as an average of E-Skin raw signal outputs for a static standing position when E-Skin sensor was at its original length and not stretched. In this experiment, we used 5 s of data to calculate the average output (i.e., baseline value). The change in electrical resistance value was calculated by Equation (5), where reference voltage for 4096 ADU is 3.3 V and the current supplied is 5 mA.

(5) $$Y=\frac{X}{4096}\times 3.3÷0.005$$

Linear regression was applied to model the mapping between E-Skin and ViMove outputs because our results showed that the skin stretch increased as the lumbar–pelvic angle increased (during flexion or extension), and there was a potential linear relationship between the outputs of E-Skin and ViMove. The linear relationship between the two variables can be presented as formula in Equation (6). Let X be the independent variable (E-Skin outputs in normalised resistance value), and Y be the dependent variable (ViMove outputs). In this work, we used the quadratic loss function in Equation (7) to calculate the loss of the model and least-squares method in Equations (8) and (9) to minimize the loss. $p_{i}$ refers to the points on the regression line. $\bar{x}$ and $\bar{y}$ are the mean values of input X and the desired output Y.

(6) Y=mX+c

(7) $$L\left(x\right)=\;\sum_{i=1}^{n}{\left(y_{i}-p_{i}\right)}^{2}$$

(8) $$m=\frac{\sum_{i=1}^{n}\left(x_{i}-\bar{x}\right)\left(y_{i}-\bar{y}\right)}{\sum_{i=1}^{n}{\left(x_{i}-\bar{x}\right)}^{2}}$$

(9) $$c=\;\bar{y}-m\bar{x}$$

The model is trained based on 10 times of each lumbar–pelvic movements’ data and tested on the other 5 times lumbar–pelvic movements’ data. Mean absolute error (MAE) of the two measurement systems (E-Skin sensor and ViMove) is used to measure the angular displacements.

To further illustrate the statistical significance, a two-tailed test was added and the *p*-Value with a significance level of 0.01 was considered to determine the statistical significance of our results. This paper uses the 15 times of each lumbar–pelvic movement to calculate the *p*-Value. However, the data quantities of each participant are different because they conducted the experiments at different speed.

The data analysis of each experiment is described in the following subsections.

#### 3.3.1. Flexion Result Analysis

**Figure 7:**
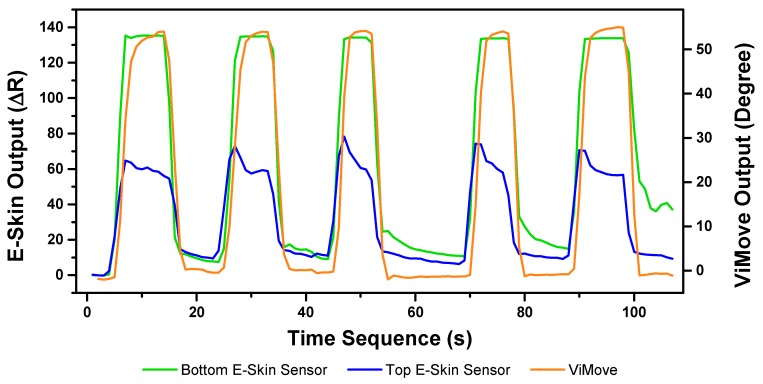
Data Outputs of Flexion.

The E-Skin measurement for the flexion movement is shown in Figure 7. The *y*-axis on the left represents the E-Skin sensor output. The *y*-axis on the right represents the ViMove output. The unit of ViMove output is the degree of anatomical angles and the unit of E-Skin sensor output is Analog-to-Digital Units (ADU) [77]. The *x*-axis represents time and the unit is second. The orange line represents the outputs of ViMove. The green line represents the outputs of the bottom E-Skin sensor and the blue line represents the outputs of the top E-Skin sensor.

For each flexion, as the orange line shows, the bending range of the participant is from 0 degrees (standing straight) to roughly 55 degrees (bending to the maximum position). The participant stayed at the maximum forward flexion position for approximately 10 s. In this work, we instructed the participants to self-report standing straight when they are at their tallest position. It can be seen that outputs of both bottom (green line) and top (blue line) E-Skin sensors data fluctuated synchronously with the changes in ViMove outputs. The E-Skin sensor outputs show a clear pattern associated with each flexion. It can also be found that both bottom and top sensors were capable of detecting the start of each flexion prior to the ViMove because the back skin stretched before the movement started. This period of time can be considered as the transition between the standing and flexion positions.

In order to analyse the response time difference between ViMove and E-Skin outputs, we calculated a threshold of ±3 degrees for ViMove outputs and a threshold of ±6.9 in resistance change for E-Skin output based on a relatively linear relationship between ViMove and the bottom E-Skin sensor outputs. We calculated the changes between sensor outputs while the participant was standing straight and performing flexion. If the changes in sensor outputs exceeded the thresholds, we considered the point as the start of a flexion. The average difference between response times of two sensors was approximately 1 s. We measured the unit of the response time difference in seconds because we computed the average of the outputs of E-Skin and ViMove in seconds as discussed earlier. The results show that E-Skin sensor can measure flexion movement faster than ViMove does. This might be due to the slimline design (maximum thickness of 5 mm) and lightweight (2 g), which allows the soft and highly stretchable E-skin sensor to adhere conformally to the curvilinear skin surface [78,79], reducing the inertial effect during movements as compared to the bulkier (100.6 mm in width, 30.4 mm in length, and 9 mm in height) and 20-fold heavier ViMove. Besides, the conformal attachment of E-skin sensors to skin surface also avoid the drifting errors that IMU-based sensor has, which allows more accurate measurement of strain [80].

We applied linear regression to model the mapping between E-Skin outputs and ViMove outputs. Our results show that R square of linear regression for the data collected from the bottom E-Skin sensor is higher than the R square for the data from the top E-Skin sensor. The R square of linear regression using both sensors is 0.2% higher than the R square for the bottom E-Skin sensor. The results are the same considering the adjusted R square. Additionally, the *p*-Value for both top and bottom E-Skin sensor outputs is statistically highly significant (*p* < 0.001). This indicates that both top and bottom E-Skin outputs have relatively linear correlations with the trunk flexion angles, but the bottom ones are more appropriate for flexion angle measurement. The details of comparison results are shown in Table 3. The data quantities of calculating these *p*-Values of flexion are 363.

Table 2 results also show that the E-Skin sensor can be used for detecting the flexion with high accuracy based on clear and repeating patterns in the output. It also shows that the bottom E-Skin sensor’s position is the suitable position for measuring the flexion. This experiment was conducted on one participant, the mapping model can be improved by collecting data from a larger populated group and considering adding more features such as age, height, weight, BMI, and skin characteristics.

As the experiments progressed, the differences between the maximum outputs of top and bottom E-Skin sensors and the participant’s maximum forward flexion angle for each flexion became relatively small. However, the differences between minimum outputs of top and bottom E-Skin sensors and the participant’s standing straight angle increased after each flexion. In Figure 2, we had conducted 1500 times cyclic strain test and the result showed that after 1000 times strain training, the sensor performance appeared to be consistent. However, the drift appeared when the sensor was mounted on individuals, and this had been consistently occurring. We believe that this is an issue with the adhesives when sticking on individuals with different skin types, particularly dry skin, sweaty skin, and individuals with a lot of dead skins and this causes the sensor to not mount properly and slide against the skin when being stretched.

**Figure 8:**
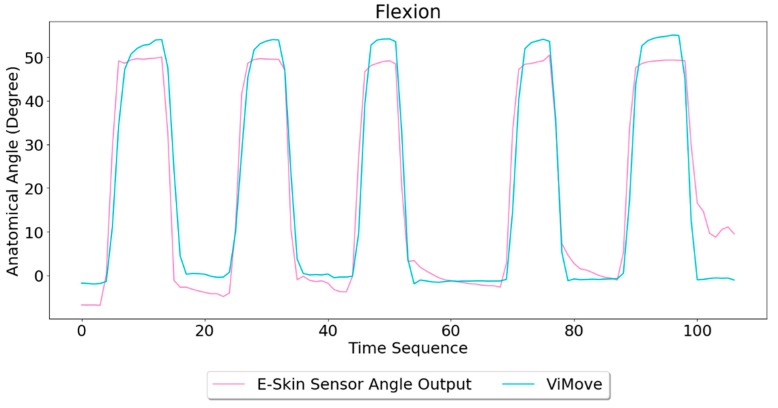
Anatomical Angle Outputs Comparison (Flexion).

Based on the model, the E-Skin sensor anatomical angle outputs of flexion are generated as shown in Figure 8. It can be seen that E-Skin sensor angle outputs are very close to the actual angles (ViMove outputs). The MAE of the two measurement systems is 5.105 degrees.

#### 3.3.2. Extension Result Analysis

**Figure 9:**
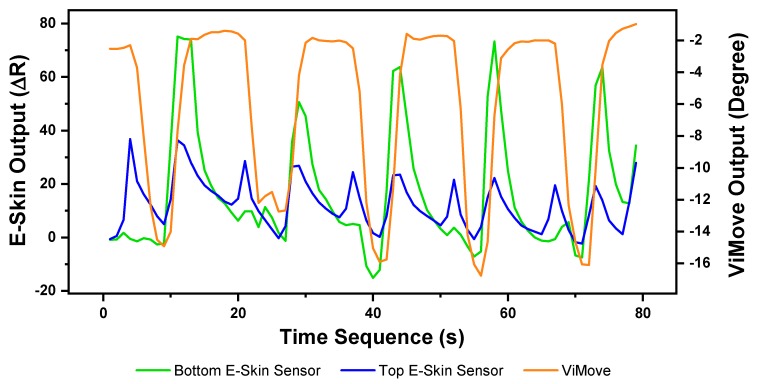
Data Outputs of Extension.

The results of our experiment to detect and measure extension are presented in Figure 9. As the figure shows, each extension started at 0 degrees (standing straight) and ended at around −12 degrees (extend to maximum position). Unlike the results of flexion, the participant did not extend to the maximum position for every extension.

The lowest extension angle is from −10 degrees to −17 degrees. The reason is because the extension requires an individual to put their head back and look at the ceiling. During this movement, an individual might only put their head back rather than extending the lumbar spine (low back) to the maximum position. Results show E-Skin sensor outputs do not closely fluctuate in line with the changes of ViMove outputs but there is a clear pattern for each extension. There are two regular spikes of top E-Skin sensor outputs (blue line) at the start and the end of each extension. There is also a notable variation in the output of the bottom E-Skin sensor when moving from the extension position to a standing straight position. The peak point of the bottom E-Skin sensor’s outputs at the second extension is lower than the other extensions which could be related to the extension angle. Similar patterns can also be found in other extensions. Additionally, the highest value of the bottom E-Skin sensor outputs changes according to the participant’s maximum extension angle in each extension. These regularities in data patterns can be used to detect an extension based on E-Skin outputs [81].

Similar to other movements, linear regression was applied to analyse E-Skin and ViMove outputs. As shown in Table 4, the R square of linear regression for extension is considerably lower than results for flexion. This can be attributed to the following reasons. First, the relationship between the extension’s angle and the lower back area skin deformation is not linear. Second, the two positions (top and bottom) may not be the optimal positions for detecting the extension. The other possible positions could be tested to increase the accuracy of measurement. Despite this, the results still show that the *p*-value of top E-Skin outputs is statistically significant and E-Skin sensor can be used to detect extension based on repeating patterns in the E-Skin outputs. The data quantities of calculating these *p*-Values of extension are 327.

**Figure 10:**
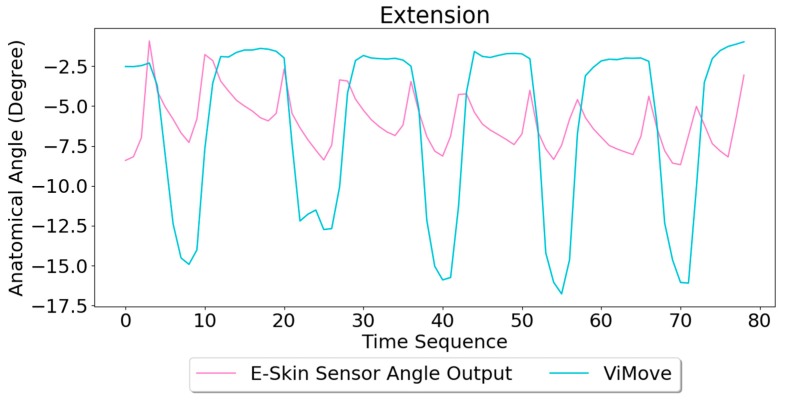
Anatomical Angle Outputs Comparison (Extension).

Based on the model, the E-Skin sensor anatomical angle outputs of extension are generated as shown in Figure 10. The MAE of the two measurement systems is 4.509 degrees. Although the MAE is lower than flexion, the angle outputs of E-Skin sensors for extension are not close to the actual angle outputs (ViMove outputs), as expected based the linear regression analysis.

#### 3.3.3. Anterior and Posterior Pelvic Tilt Result Analysis

**Figure 11:**
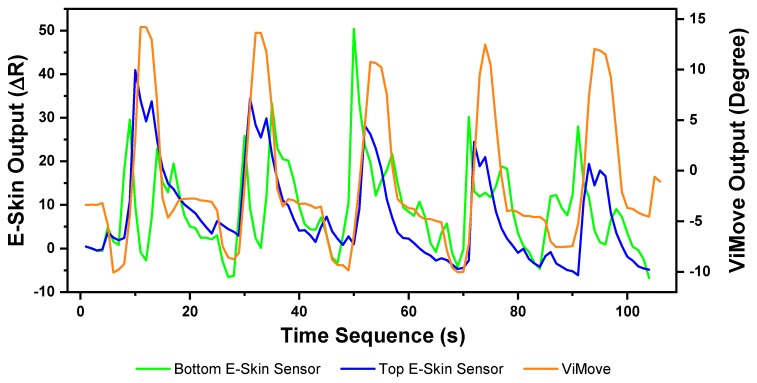
Data Outputs of Pelvic Tilt.

The pelvic tilt consists of anterior and posterior pelvic tilt. The data outputs of the anterior and posterior pelvic tilt are shown in Figure 11. The figure shows that there is an obvious rise and fall patterns in both sensor outputs when the participant performs pelvic tilt. Yet, the data patterns for pelvic tilt are different compared to flexion and extension results. The top E-Skin sensor outputs fluctuate according to the changes of pelvic tilt, while the bottom sensor outputs show two spikes at the start and the end of each pelvic tilt.

The repeatability of E-Skin sensor output is not high enough for detecting pelvic tilt compared to the previous two experimental results. The pelvic tilt requires the participant to tilt their pelvis anteriorly and posteriorly. These movements involve excessive subtle motions that are very difficult to repeat in the exact same way each time. Skin deformation data captured by E-Skin sensor reflect these minor changes and make it hard to achieve consistent repeatability.

The results of linear regression for modelling the mapping between E-Skin outputs and ViMove outputs are similar to extension (see Table 5). However, the top E-Skin sensor outputs demonstrate a statistically highly significant correlation with the ViMove sensors outputs with an R square value at 62.9% (*p* < 0.001), while the bottom E-Skin sensor shows significantly lower results. This can be addressed by investigating and determining optimal sensor placement on the body to achieve higher sensing accuracy. The data quantities of calculating these *p*-Values of pelvic tilt are 476.

**Figure 12:**
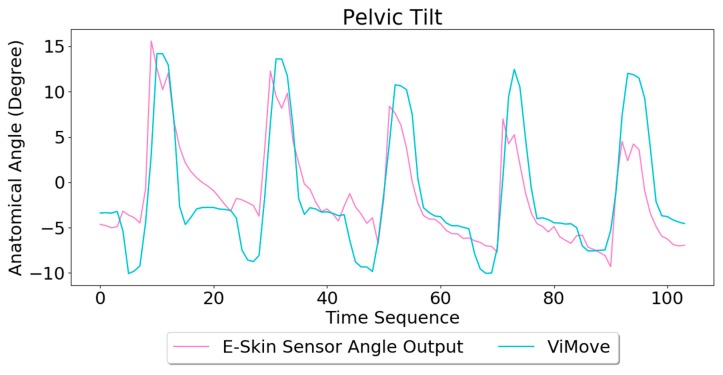
Anatomical Angle Outputs Comparison (Pelvic Tilt).

Based on the model, the E-Skin sensor anatomical angle outputs of pelvic tilt are generated as shown in Figure 12. The MAE of the two measurement systems is 3.154 degrees. The MAE of the pelvic tilt is also lower than flexion. However, it can be seen that E-Skin sensors have a better performance on measuring posterior pelvic tilt than anterior pelvic tilt. The reason for this is because the current sensor placement of bottom E-Skin sensor is not capable of fully capturing anterior pelvic tilt movement.

#### 3.3.4. Lateral Flexion and Rotation Analysis

**Figure 13:**
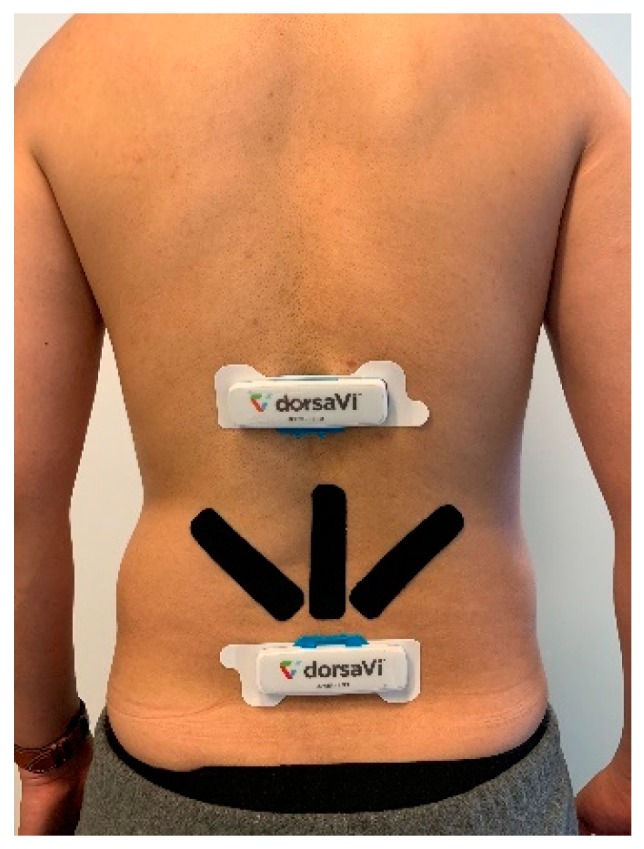
New Sensor Placement.

Lateral Flexion and Rotation are the other two standard lumbar–pelvic movements. Lateral flexion involves the torso bending movement in the lateral direction (i.e., in sideward). Rotation refers to the up-torso rotation towards the right or left side while the pelvic area stays still. These movements are more complex than flexion, extension, and pelvic tilt. Based on the E-Skin sensor settings (e.g., the number of sensors and their placement) that we used in the previous three experiments, these two movements cannot be detected with acceptable precision. Therefore, as shown in Figure 13, we had to use two additional E-Skin sensors which were positioned diagonally at both sides towards the inferior angle of the participant’s right and left scapula (ISA) while standing straight. This sensor placement is identified by the same procedures as the previous one.

##### Lateral Flexion

**Figure 14:**
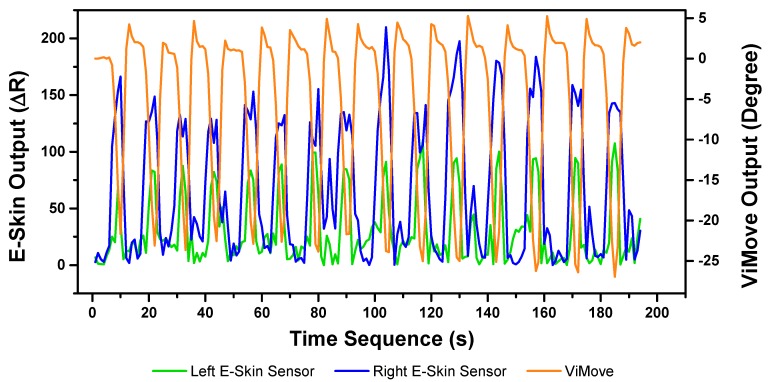
Data Outputs of Left Lateral Flexion.

**Figure 15:**
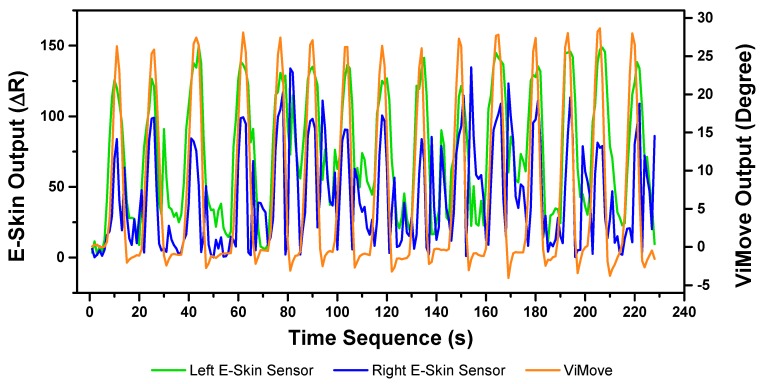
Data Outputs of Right Lateral Flexion.

The results of our experiments using E-Skin sensors and ViMove for left and right lateral flexion are shown in Figure 14 and Figure 15, respectively. The orange line still represents the outputs of ViMove. The red and purple lines represent the outputs of left and right E-Skin sensors. 

The rise and fall patterns in ViMove data shown in Figure 14 represent a single left lateral flexion movement. Figure 15 shows an opposite pattern for the right lateral flexion movement. E-Skin sensors’ outputs and ViMove outputs clearly show repeating similar patterns. Both left and right E-Skin sensor outputs fluctuate according to the changes in the lateral flexion movement. Yet, for the left lateral flexion, the range of the right E-Skin sensor outputs are higher than the left sensors’ outputs, and for the right lateral flexion, the left E-Skin sensors show higher output values. 

The linear regression results for modelling the mapping between E-Skin outputs and ViMove outputs shows that both left and right E-Skin sensor outputs have a statistically highly significant correlation (*p* < 0.001) with the actual anatomical angle (ViMove outputs). Table 6 and Table 7 show the results for left and right lateral flexion respectively. The results of the mapping models for left and right E-Skin sensors are different for left and right lateral flexion in an opposite way. The data quantities of calculating these p-Values of left and right lateral flexion are 198 and 229, respectively.

**Figure 16:**
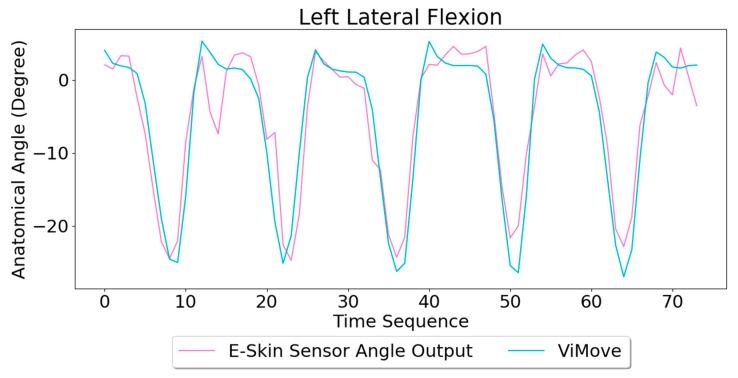
Anatomical Angle Outputs Comparison (Left Lateral Flexion).

**Figure 17:**
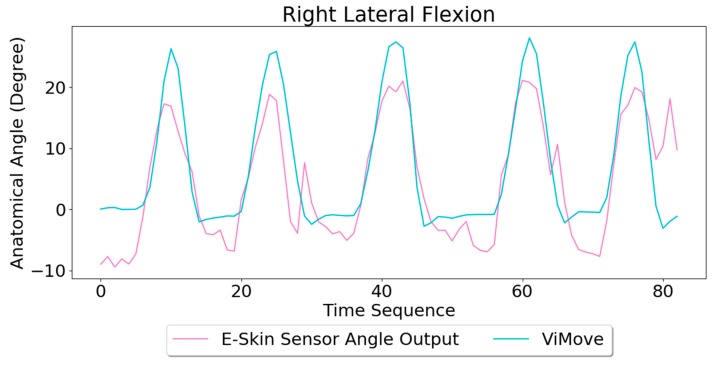
Anatomical Angle Outputs Comparison (Right Lateral Flexion).

Based on the model, the E-Skin sensor anatomical angle outputs of left and right lateral flexion are generated as shown in Figure 16 and Figure 17, respectively. The MAEs of the two measurement systems are 2.863 degrees and 5.039 degrees, respectively. The E-Skin sensor has a relatively good result on both left and right lateral flexion.

##### Rotation

**Figure 18:**
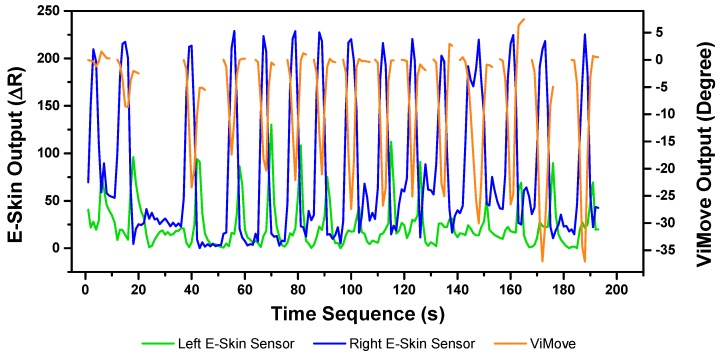
Data Outputs of Left Rotation.

**Figure 19:**
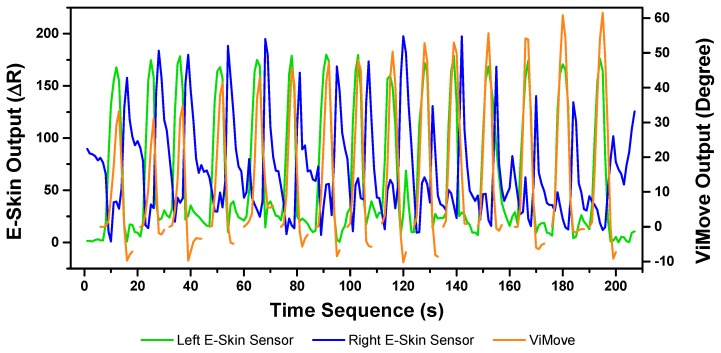
Data Outputs of Right Rotation.

The results of the left and right rotations are shown in Figure 18 and Figure 19. The ViMove outputs are represented by the orange lines. The patterns of the left and right rotation are similar to the left and right lateral flexion movements, but the difference is that there is a rise in the right E-Skin sensor outputs before the left E-Skin sensor outputs when the participant performs left rotation. In contrast, left E-Skin sensor outputs show a fall in the data values before the right sensor when the participant performs right rotation. Based on this clear and repeating trend, the rotation can be clearly detected.

The results of linear regression of modelling the mapping between E-Skin outputs and ViMove outputs are consistent with our findings based on Figure 18 and Figure 19. As shown in Table 8, the right E-Skin outputs have a stronger relationship with the actual anatomical angle (ViMove outputs) while there is a relatively weak relationship between left E-Skin outputs and the anatomical angle when the participant performs left rotation. Similar results were also found in right rotation analysis (see Table 9 summary of the comparison). The data quantities of calculating these *p*-Values of left and right rotation are 193 and 209, respectively.

**Figure 20:**
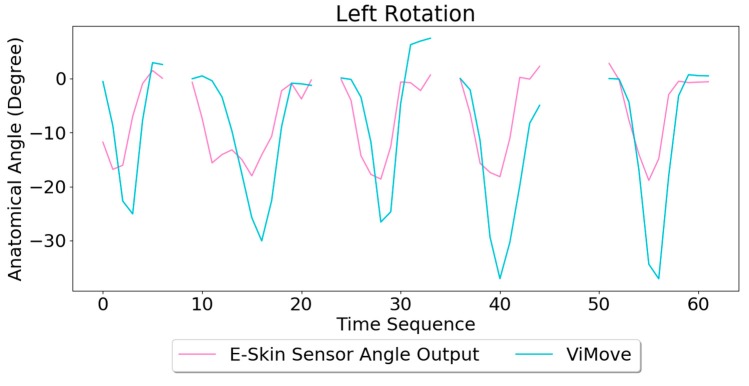
Anatomical Angle Outputs Comparison (Left Rotation).

**Figure 21:**
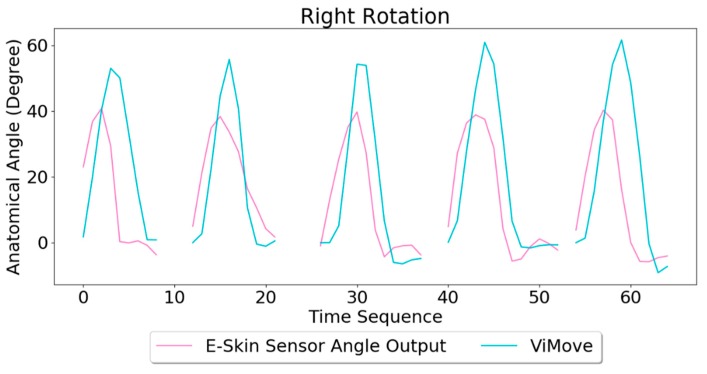
Anatomical Angle Outputs Comparison (Right Rotation).

Based on the model, the E-Skin sensor anatomical angle outputs of left and right lateral flexion are generated as shown in Figure 20 and Figure 21 respectively. The MAEs of the two measurement systems are 5.679 degrees and 7.001 degrees, respectively. The E-Skin sensors’ performances on measuring left and right rotations are relatively better than extension. However, the E-Skin sensors’ angle outputs have a larger MAE compare to left and right lateral flexion. The largest angular difference of the two measurement systems for both left and right rotations can be more than 15 degrees.

#### 3.3.5. Summary (Cross Lumbar–Pelvic Movement Analysis) 

Based on the previous analytical results, the detection and measurement of flexion, extension, and pelvic-tilt are mainly based on the top and bottom sensors. Among which, each E-Skin sensor plays different roles: (1) the bottom E-Skin sensor is important in measuring flexion angle, (2) The top E-Skin sensor is essential for the detection and measurement of pelvic-tilt. The existing sensor placement is not suitable for measure extension. In lateral flexion and rotation analysis, it can be seen that the right and left sensors are more important. According to these findings, we can draw a conclusion that vertical sensor placement on human back is suitable for detecting and measuring sagittal plane related movements. However, different sagittal plane movement may require different vertical positions for sensors. On the other hand, sloping or horizontal sensor placements are more suitable to detect and measure the movements which are performed in coronal and transverse planes.

## 4. Discussion

### 4.1. Principle Results

In this study, the potential of using E-Skin sensors (resistive flex sensor) to detect and measure lumbar–pelvic movements was identified through a detailed classification and comparison of existing human motion sensing technologies. Our experimental results confirmed that these stretchable and highly sensitive sensors were capable of measuring five lumbar–pelvic movements and could be used for detecting these movements based on their obvious repeating patterns. The performance of E-Skin sensors was evaluated against the ViMove outputs as a clinically tested gold standard. We applied linear regression to model the mapping of E-Skin outputs to the actual movement angles captured by ViMove. We found that E-skin sensor was capable of measuring simple anatomical joint movements such as trunk flexion. The results also showed the E-Skin had a quicker response to movement changes than the IMU-based sensor systems. It was also able to measure flexion movement faster than ViMove.

Overall, our results show that E-Skin sensors have the potential for continuous detection and measurement of lumbar–pelvic movements in out-of-lab settings. They can be used as part of low back pain management and monitoring systems to assist individuals during their rehabilitation.

### 4.2. Study Limitations

In this study, we were able to model the mapping of the E-Skin outputs to actual anatomical joint angles in lumbar–pelvic movements, but this mapping can be further improved. The aim of this study is to explore the potential of using E-Skin sensor as an alternative for the lumbar–pelvic movement detection and measurement. Only limited numbers of participants were recruited in this study. Considering the individual variability in skin deformations when establishing the mapping model, different individuals may have different skin characteristics (e.g., loose and tight), age, height, weight, and BMI. These personalised factors need to be taken into consideration during the modelling. More experiments with different participants who have different skin characteristics and back shapes are needed to collect a wide range of real data for establishing a generic robust mapping model to accurately detect and measure different body movements.

## 5. Conclusions and Future Work

E-Skin sensors have become a new trend in the field of wearable sensors and body motion detection [82]. However, current studies use the E-Skin sensors for measuring movements such as the motion of the throat muscles [83] or fingers [64]. There is a lack of studies that consider using E-Skin sensors for detecting and measuring lumbar–pelvic movements. In the meantime, the need for continuous and out-of-hospital monitoring and management of low back pain propels the use of low-cost and comfortable wearable sensors.

The human body is not a rigid and plain surface, and the lumbar spine shape varies from individual to individual. Traditional IMU-based sensors (e.g., ViMove) are attached to the human skin by using medical tapes [42]. Because IMU-based sensors are not stretchable, there may be deviations from the original sensor placement after the individual performs body movements which may lead to measurement errors [70]. Compared to the IMU-based sensors, E-Skin sensors are stretchable and can be attached comfortably to different types and shapes of human skin. Comparing to vision-based sensing system, E-Skin sensor also was identified to be much more comfortable for long-term monitoring and does not have the soft tissue artefacts. These characteristics makes it a suitable alternative for continuous out-of-hospital monitoring of such movements. The experimental results of this paper prove that the vertical placement of the E-Skin sensors on human back is capable of measuring sagittal plane related movements such as flexion. The horizontal/inclined placements of E-Skin sensors on human back is suitable for measuring coronal and transverse plane related movements such as lateral flexion and rotation. Therefore, the E-Skin sensor had great potential to detect the features of different lumbar–pelvic movements and measure anatomical joint angles.

For future work, we intend to conduct the experiments on a larger population. We also plan to consider different combinations of the multiple sensor placements. Because skin deformations do not provide any information about the dimension (i.e., the directions of body movements in three-dimensional space), multiple sensors with the optimal sensor placements may provide valuable information for selecting more effective data fusion techniques to model the three-dimensional movements. Different machine learning algorithms and data fusion methods such as SVM, Decision Tree, and kNN [84] need to be explored and tested for establishing a robust and accurate mapping model. We also plan to also extend our work to other types of body movements including knee bending, ankle rotation, and neck flexion.

## Figures and Tables

**Table 1:** Comparison of Existing Human Motion Sensing Systems.

	Vison-Based System		Inertial Measurement Unit			Flex Sensor	
Technology	Marker-based Systems	Marker-less Systems	Single IMU	Dual IMUs	Multiple Sensory	Optical Fiber	Conductive Material
Products	Vicon; CODA; Optotrak	Kinect; KinaTrax	Lumo Lift; Upright	ViMove; Valedo	ZiShi	[34,35]	Strechsense; E-Skin
Measurements	3D Coordinates					Structural beams	Skin Deformation
Mobility	In-lab	Limited Range	Out-of-lab				
Pros	High Precision and Accuracy; Gold Standard	Easy to Setup; Less Expensive	Low Cost; Continuous out-of-lab monitoring; Easy to Setup	Continuous out-of-lab monitoring; Measure complex human motions	Continuous out-of-lab monitoring; Multi-sensory data fusion	Garment-integrable; Long-term monitoring	Long-term Monitoring; Lower Cost; Measure complex activity
Cons	In-Lab Setting; Expensive; Sensitive to lighting conditions; Soft tissue artefacts	Limited Range; Sensitive to lighting conditions	Measure simple human motions; Lower Accuracy	Expensive; Bulky	Soft tissue artefacts; Non-washable; Complex calibration process	Measure simple activity; Complex setting	Durability; Complex modelling process

**Table 2:** Comparison of E-Skin sensor and ViMove.

	E-Skin	ViMove
Physical Property	Soft	Rigid
Measurement	Skin Deformation	3D Coordinates
Connectivity	Bluetooth 4.0 (BLE)	2.3 Ghz Frequency
Data Collection Device	Smartphone/Tablet	PC
Clinical Validation	NO	YES
Battery Life	1 week	24 h
Price	AUD $10~15 each module * (* not yet available for public sale)	AUD $10,000 (Hardware and Software)

**Table 3:** Linear regression comparison for flexion.

Independent	R	R^2^	Adjusted R^2^	Significance (2-Tailed)
Top E-Skin Outputs	0.843	0.711	0.708	*p* < 0.001
Bottom E-Skin Outputs	0.954	0.910	0.909	*p* < 0.001
Both	0.955	0.912	0.911	

**Table 4:** Linear regression comparison for extension.

Independent	R	R^2^	Adjusted R^2^	Significance (2-Tailed)
Top E-Skin Outputs	0.318	0.101	0.090	0.004
Bottom E-Skin Outputs	0.183	0.033	0.021	0.107
Both	0.320	0.102	0.079	

**Table 5:** Linear regression comparison for pelvic tilt.

Independent	R	R^2^	Adjusted R^2^	Significance (2-Tailed)
Top E-Skin Outputs	0.793	0.629	0.625	*p* < 0.001
Bottom E-Skin Outputs	0.128	0.016	0.007	0.196
Both	0.796	0.633	0.626	

**Table 6:** Linear regression comparison for left lateral flexion.

Independent	R	R^2^	Adjusted R^2^	Significance (2-Tailed)
Left E-Skin Outputs	0.817	0.667	0.665	*p* < 0.001
Right E-Skin Outputs	0.832	0.692	0.690	*p* < 0.001
Both	0.893	0.798	0.796	

**Table 7:** Linear regression comparison for right lateral flexion.

Independent	R	R^2^	Adjusted R^2^	Significance (2-Tailed)
Left E-Skin Outputs	0.798	0.637	0.635	*p* < 0.001
Right E-Skin Outputs	0.496	0.246	0.243	*p* < 0.001
Both	0.804	0.646	0.643	

**Table 8:** Linear regression comparison for left rotation.

Independent	R	R^2^	Adjusted R^2^	Significance (2-Tailed)
Left E-Skin Outputs	0.110	0.012	0.005	*p* < 0.001
Right E-Skin Outputs	0.747	0.558	0.555	*p* < 0.001
Both	0.768	0.590	0.584	

**Table 9:** Linear regression comparison for right rotation.

Independent	R	R^2^	Adjusted R^2^	Significance (2-Tailed)
Left E-Skin Outputs	0.843	0.710	0.709	*p* < 0.001
Right E-Skin Outputs	0.483	0.233	0.229	*p* < 0.001
Both	0.843	0.711	0.708	
